# Associations Between Area Deprivation Index and the Time to Presentation of Scaphoid Fractures

**DOI:** 10.1016/j.jhsg.2025.100932

**Published:** 2026-01-19

**Authors:** Aidan M. Lynch, Muhammad H. Hamdan, Jordan Bauer, Rohan V. Rajan, Rajendra Singh, Joel V. Ferreira, Anthony Parrino, Craig M. Rodner

**Affiliations:** ∗ Department of Orthopedic Surgery, University of Connecticut, Farmington, CT; † Department of Orthopedic Surgery, University of Connecticut, Farmington, CT

**Keywords:** Area deprivation index, Nonunion, Scaphoid fractures, Social determinants of health

## Abstract

**Purpose:**

Scaphoid fractures are the most common carpal fractures and are prone to nonunion because of their retrograde blood supply. Delayed diagnosis or treatment increases the risk of nonunion and progression to scaphoid nonunion advanced collapse. Social determinants of health, measured by the area deprivation index (ADI), may contribute to such delays. This study examines the association between ADI, time to presentation, and the presence of nonunion at initial evaluation.

**Methods:**

A retrospective chart review identified 168 patients with suspected scaphoid fractures between January 1, 2018 and June 30, 2025. State-level ADI scores were grouped into the following terciles: least-deprived (LDT), intermediately deprived (IDT), and most deprived (MDT). Patients presenting more than 5 years after injury or without confirmed fractures were excluded, leaving a total of 107 patients. Independent *t* tests compared the mean time from injury to presentation across ADI terciles, χ^2^ tests compared the nonunion rates at presentation, and binomial regression assessed whether ADI predicted scaphoid nonunion.

**Results:**

The mean time from injury to presentation increased with deprivation: LDT = 16.9 ± 23.5 days, IDT = 55.1 ± 97.9 days, and MDT = 173.5 ± 364.5 days. Both MDT and IDT patients presented significantly later than LDT patients (*P* = .0146 and *P* = .0315, respectively) and had a higher prevalence of nonunion at presentation (*P* = .025). The ADI independently predicted scaphoid nonunion, with each unit increase in state ADI associated with 23.8% higher odds of nonunion (*P* = .029).

**Conclusions:**

Patients from socioeconomically deprived communities experienced significantly longer delays in presentation for scaphoid fractures and a higher incidence of nonunion, which may increase the risk of long-term complications such as scaphoid nonunion advanced collapse wrist arthritis. These findings highlight the importance of addressing neighborhood-level disparities to increase access to treatment in time-sensitive injuries such as scaphoid fractures.

**Type of study/level of evidence:**

Prevalence IIIb.

Scaphoid fractures are the most common carpal fractures, predominantly impacting young, active individuals, particularly men; this injury often occurs as a result of a fall on an outstretched arm.^1^, ^2^, ^3^, ^4^ The bone’s unique blood supply and anatomical position make it susceptible to nonunion and avascular necrosis if not properly treated.^2^^,^^5^^,^^6^ Nonunion can lead to scaphoid nonunion advanced collapse (SNAC) and subsequent degenerative arthritis of the wrist of patients, with proximal pole fractures particularly prone to avascular necrosis and degenerative changes.^7^ Therefore, early diagnosis and appropriate management of scaphoid fractures are crucial to preventing nonunion and its subsequent complications.

Initial diagnosis of suspected scaphoid fractures, such as in people with snuffbox pain after a fall on an outstretched wrist, is determined by clinical examination and radiographic imaging. Some scaphoid fractures may produce nonrevealing initial radiographic imaging, which, in the presence of snuffbox tenderness, should be treated with initial immobilization, followed by repeat imaging a week or two later; if plain films remain inconclusive, but snuffbox tenderness persists. Advanced imaging, such as magnetic resonance imaging (MRI) or computed tomography (CT) scan, should be considered to assess for an occult scaphoid fracture.^4^^,^^6^

Individuals without insurance or on public health insurance routinely experience delays in receiving care and present with more advanced conditions for orthopedic problems.^7^, ^8^, ^9^, ^10^, ^11^ While this association is strong, insurance status does not fully encapsulate a patient’s social determinants of health (SDOH).^12^ One way to more accurately measure the SDOH is through the area deprivation index (ADI), which stratifies neighborhoods by adverse social exposure, taking into account income, education, employment, and housing quality.^13^ Although there are studies examining insurance status in hand surgery and ADI, to our knowledge, there is no literature studying how SDOH may affect a patient’s presentation following a scaphoid fracture. This study aims to explore the relationship between ADI score and time to presentation and development of nonunion in scaphoid fractures.

## Materials and Methods

This study is a retrospective chart review of patients who were seen at a single academic medical center for scaphoid fractures with an International Classification of Diseases, Ninth and Tenth Revisions (ICD-9 or ICD-10) code encompassing scaphoid fracture diagnoses between January 1, 2018 and June 30, 2025. Patients were excluded if further imaging, such as an MRI or CT scan, did not reveal a scaphoid fracture. Patients who presented more than 5 years after injury were excluded to minimize bias from extreme outliers. These cases, some presenting up to 50 years after injury, were considered atypical and not representative of contemporary diagnostic or treatment delays. The Institutional Review Board approval was obtained, and data were coded for Health Insurance Portability and Accountability Act compliance.

Data collected included the following: nine-digit zip code, age at presentation, time from injury to presentation, and presence of radiographic nonunion. Imaging protocols were standardized at our institution, with all patients receiving wrist radiographs and advanced imaging (MRI or CT) when initial films were inconclusive. Using the nine-digit zip code, a state ADI score was assigned to each patient using the University of Wisconsin School of Medicine’s ADI tool.^13^ With respect to demographic information, ADI is a comprehensive tool that has been demonstrated to be more predictive of SDOH than individual-level measures such as race or ethnic background.^14^ Thus, age was the only individual-level measurement analyzed between groups to account for potentially confounding variables. Patients were categorized into the following three groups based on these scores: least-deprived tercile (LDT), intermediately deprived tercile (IDT), and most deprived tercile (MDT) for the state ADI scores.^15^ Using IBM SPSS software, independent *t* tests and Wilcoxon signed-rank tests were run between ADI terciles and time to presentation, a χ^2^ test was used to evaluate the prevalence of nonunions, and a binomial regression was used to determine if ADI predicts scaphoid nonunion. Significance was defined as *P* < .05.

## Results

A total of 168 patients were identified by ICD-9 and -10 codes for scaphoid fracture who presented between January 1, 2018 and June 30, 2025. From this cohort, 52 were excluded for not having a scaphoid fracture after advanced imaging, and 9 were excluded for presenting after 5 years, giving a final study population of 107 patients. Of these patients, 34 were in the LDT, 27 in the IDT, and 46 in the MDT. The final study population had an average age of 29.0 ± 15.4 years. The LDT had an average age of 31.2 ± 17.2 years, the IDT had an average age of 28.8 ± 17.1 years, and the MDT had an average age of 27.5 ± 12.8 years. Analysis of variation was not significant (*P* = .568).

**Figure 1:**
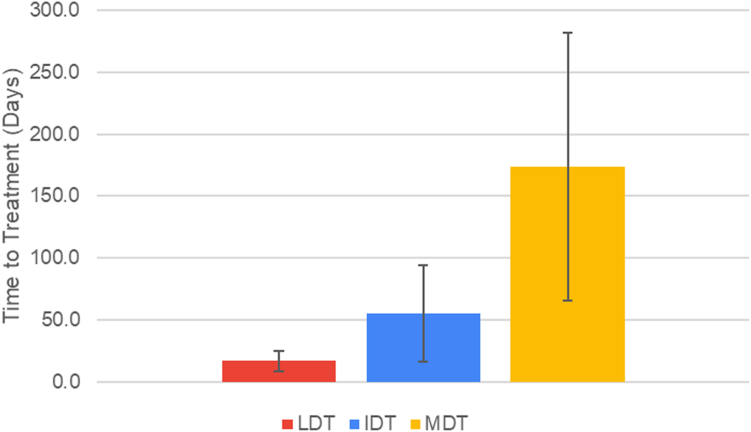
Mean time to treatment stratified by ADI. Error bars correspond to a 95% CI.

The LDT had an average time to presentation of 16.9 ± 23.5 days. The IDT had an average time to presentation of 55.1 ± 97.9 days, and the MDT had an average time to presentation of 173.5 ± 364.5 days (Table 1, Fig. 1). The MDT had a longer time to presentation than the LDT (*P* = .015), as did the IDT (*P* = .032). The MDT did not have a statistically longer time to treatment than the IDT (*P* = .104). Although normality testing did not indicate significant deviation (*P* > .05 for every tercile), the large standard deviation and visual skew suggest that median and interquartile range (IQR) may better represent the data. The LDT had a median time to presentation of 6.0 (IQR = 17.0) days. The IDT had a median time to presentation of 20.0 (IQR = 53.0) days, and the MDT had a median time to presentation of 29.5 (IQR = 138.5) days (Table 1). Wilcoxon signed-rank test testing showed that the MDT had a longer time to presentation than the LDT (*P* < .001), and the IDT had a longer time to presentation than the LDT (*P* = .001). The MDT did not have a statistically significant longer time to presentation than the IDT (*P* = .464).

**Table 1:** Mean and Median Time From Injury to Presentation for Each ADI Tercile

ADI Tercile	N	Mean Time From Injury to Presentation in D (SD)	Median Time From Injury to Presentation in D (IQR)
LDT	34	16.9 (±23.5)	6.0 (17.0)
IDT	27	55.1 (±97.9)	20.0 (53.0)
MDT	46	173.5 (±364.5)	29.5 (138.5)

**Figure 2:**
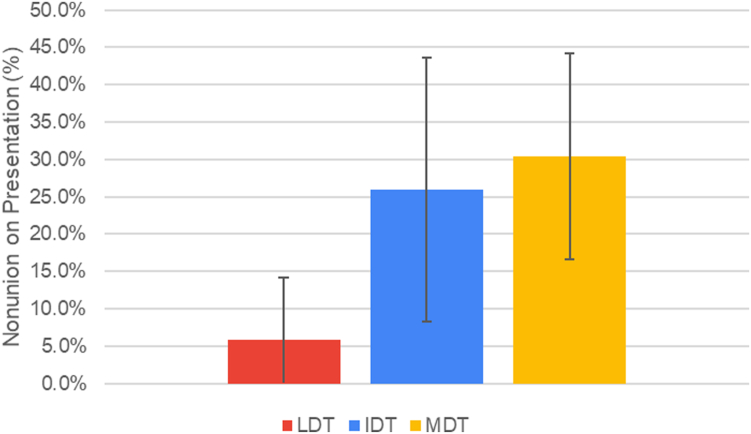
Incidence of scaphoid nonunions on initial presentation for each ADI tercile. Error bars correspond to a 95% CI.

Upon initial presentation, 5.9% of the LDT patients presented with radiographic evidence of a scaphoid nonunion, which was significantly less (*P* = .025) than the 25.9% of scaphoid nonunions in the IDT and the 30.4% of the MDT (Table 2, Fig. 2)

**Table 2:** Scaphoid Nonunions at the Time of Presentation for Each ADI Tercile

ADI Tercile	N	Nonunion on Presentation (%)
LDT	34	2 (5.9)
IDT	27	7 (25.9)
MDT	46	14 (30.4)

**Figure 3:**
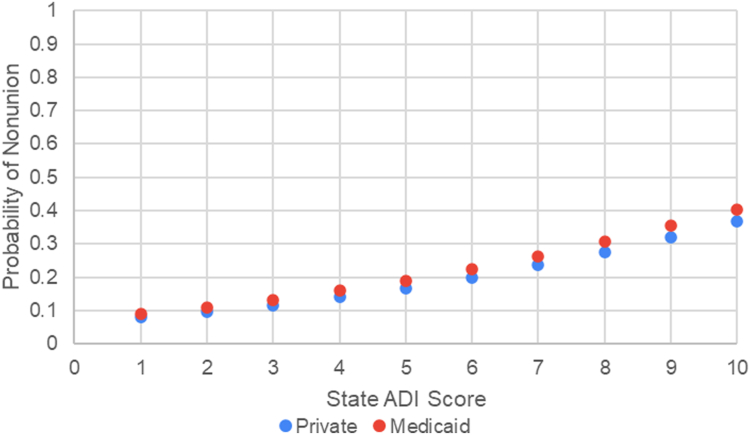
Predicted probability of nonunion by state ADI score for private and Medicaid insurance.

A binary logistic regression was conducted in SPSS to examine the association between state ADI score and the likelihood of presenting with a scaphoid nonunion, controlling for age and insurance status. The model was statistically significant (χ^2^(1) = 4.754, *P* = .029), indicating that ADI significantly predicted nonunion status, whereas age (*P* = .812) and insurance status (*P* = .765) did not significantly predict nonunion. The logistic regression coefficient for state ADI was B = 0.213 (*P* = .029), corresponding to an odds ratio of 1.238, meaning that each one-unit increase in ADI is associated with a 23.8% increase in the odds of nonunion. The model predicts a 6.8-fold increase in the odds between the lowest and highest ADI levels (Fig. 3).

**Figure 4:**
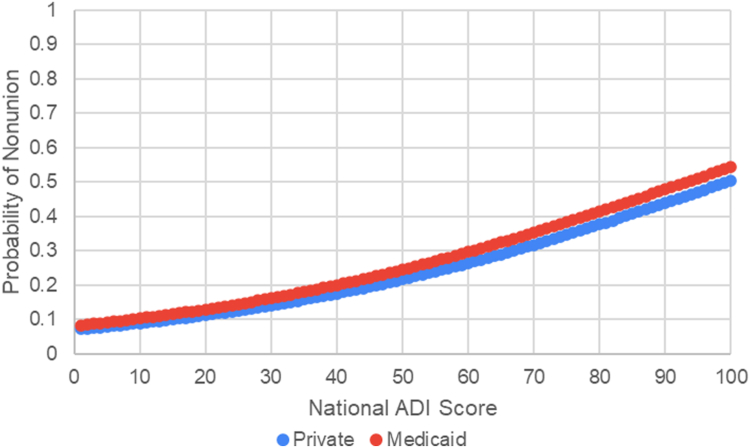
Predicted probability of nonunion by national ADI score for private and Medicaid insurance.

A second binary regression was conducted to examine the association between national ADI score and the likelihood of presenting with a scaphoid nonunion, controlling for age and insurance status. The model was statistically significant (χ^2^(1) = 4.254, *P* = .039), indicating that ADI significantly predicted nonunion status, whereas age (*P* = .885) and insurance status (*P* = .746) did not significantly predict nonunion. The logistic regression coefficient for national ADI was B = 0.026 (*P* = .039), corresponding to an odds ratio of 1.026, meaning that each one-unit increase in national ADI (scored from 1 to 100) is associated with a 0.26% increase in the odds of nonunion. The model predicts a 13.1-fold increase in the odds between the lowest and highest ADI levels (Fig. 4).

## Discussion

This retrospective study spanning nearly a decade leveraged state ADI data to examine the relationship between ADI and time to presentation in scaphoid fractures. Across 107 patients, these data provide evidence that patients with scaphoid fractures in the most privileged communities (LDT) presented considerably more promptly than patients in more disadvantaged communities (IDT and MDT). In other words, individuals from disadvantaged communities presented with a more delayed time to initial treatment than those from more privileged communities, and a considerably higher rate of nonunion on initial presentation. Nonunions generally lead to worse outcomes because of the development of rates of SNAC wrist arthritis, chronic pain, and subsequent degenerative arthritis of the wrist.^6^^,^^16^, ^17^, ^18^ Reducing the barriers to care that delay the time to treatment of patients from more deprived communities is essential to optimize outcomes across all patient populations.

Scaphoid fractures are categorized by their location—proximal pole, waist, or distal pole. A retrograde blood supply, distal to proximal, from the dorsal carpal branch of the radial artery perfuses the scaphoid bone, which leaves the proximal pole and waist fractures at a greater risk of nonunion. Proximal pole fractures account for 25% of cases, waist fractures, 65%, and distal pole fractures, 10%. Fracture distance from the distal blood supply directly relates to longer recovery times and the need for surgical repair.^19^^,^^20^ Because of the anatomical risk of nonunion of the proximal pole fracture, minimizing all delays in presentation and care is paramount.

Delays in access to care, such as those associated with higher ADI terciles, can directly influence the treatment options available for patients. Although nonsurgical cast immobilization and percutaneous internal screw fixation are the standard techniques for acute and stable scaphoid fractures, delayed presentation increases the risk of nonunions, resulting in more invasive procedures. Nonunion fractures typically require an open fixation with bone grafts—vascularized or nonvascularized.^6^ If the surgical fixation fails to unionize or the treatment delay is more significant, it may lead to the development of SNAC wrist arthritis. The longer the SNAC wrists are left untreated, the further the arthritic degeneration occurs, leading to the need for salvage reconstructive procedures and arthrodesis, considerably limiting wrist motion and grip strength compared with original baseline status.^6^ Per our study, a delay in treatment is directly related to a higher ADI tercile, which suggests that patients in higher ADI terciles are more likely to need higher-risk surgical interventions.

Several studies have examined the relationship between orthopedic care and socioeconomic status (SES). Lower SES has been consistently associated with higher rates of orthopedic injury, particularly high-energy and violent trauma.^21^, ^22^, ^23^, ^24^, ^25^ Breslin et al^26^ examined the impact of the SDOH on the rates of intentional orthopedic trauma, identifying a link between the mechanism of traumatic orthopedic injury and SDOH risks. Patients from disadvantaged backgrounds, characterized by lower income, public insurance, educational deficiencies, and social deprivation, are more likely to experience delays in diagnosis and treatment, higher complication rates, and poorer functional and mental health at presentation; these disparities are particularly pronounced in rural and low-income urban populations, where mortality and adverse outcomes after orthopedic trauma are elevated.^26^, ^27^, ^28^ Patient-level SDOH factors such as unreliable transportation, housing instability, and financial hardship directly limit the ability to attend appointments and adhere to treatment plans, resulting in worse patient-reported outcomes and increased risk of complications after fracture care. In addition, patients with public insurance and high deprivation face difficulties scheduling appointments, longer wait times, and a more limited selection of available practices.^9^^,^^29^^,^^30^ In addition, previous literature has used ADI to examine the impact of SES on outcomes in outpatient hand surgery, finding an inverse relationship between SES and functional scores.^31^ Looking specifically at the scaphoid, Garala et al^32^ conducted a retrospective study in the United Kingdom, finding that patients with the lowest SES had nearly double the incidence of scaphoid fractures compared with their higher-SES peers.

Delayed diagnosis and treatment of scaphoid fractures have been associated with poorer outcomes.^33^ Given this well-established link, pathways that connect at-risk patients with prompt and appropriate management should aim to target patients from disadvantaged backgrounds. In addition to improving provider awareness, early radiographs, and, when indicated, advanced imaging and targeted outreach have been shown to improve outcomes. Typical algorithms for suspected scaphoid fractures emphasize early MRI if no fracture is seen on X-ray or CT scan and if a fracture appears nondisplaced on X-ray to minimize the time to effective treatment.^6^ Murthy^34^ examined the role of advanced imaging in scaphoid fractures, finding that early MRI was both cost and clinically effective, resulting in improved patient outcomes and lower overall cost. The cost savings of early interventions are especially important for safety-net institutions caring for uninsured or underinsured patients, as recent Medicaid cuts and under-reimbursement contribute to financial instability.^35^ Furthermore, expedited referrals or “fast-track pathways” from the emergency department to the operating room for hip surgeries within the first 24 hours of the injury have been shown to reduce mortality and complications. Such protocols can further reduce disparities and delayed presentation as these patients are cared for immediately.^36^ Future multicenter studies aimed at improving time to presentation, such as more streamlined referral paths, early imaging, or investing in community outreach, are warranted.

This study has several limitations. First, as a retrospective chart review, our study relies on the accuracy and completeness of medical records. Second, although ADI is a validated model of assessing a patient’s SDOH, it may not directly capture all individual-level socioeconomic factors, such as transportation and access to medical care. Third, our study only included 107 patients, which may limit the statistical power to detect notable associations and reduce the precision of effect estimates. Fourth, some patients may have received initial assessment or imaging elsewhere, which could bias findings toward longer presentation delays. Fifth, specific treatment options and long-term outcome measurements were not collected in this cohort. Future studies would significantly benefit from comparing these variables between ADI groups. Finally, as this analysis was conducted at a single institution, findings may not be as generalizable as those collected from multiple systems or geographic regions.

This study provides evidence that patients from disadvantaged neighborhoods experience longer delays in scaphoid fracture presentation and have a higher incidence of scaphoid nonunion on presentation. Future efforts need to be made to address these health disparities.

## Conflicts of Interest

No benefits in any form have been received or will be received related directly to this article.

## References

[1] Almigdad A., Al-Zoubi A., Mustafa A. (2024). A review of scaphoid fracture, treatment outcomes, and consequences. Int Orthop.

[2] Xiao M., Welch J.M., Cohen S.A., Kamal R.N., Shapiro L.M. (2023). How is scaphoid malunion defined: A systematic review. Hand N Y N.

[3] Hove L.M. (1999). Epidemiology of scaphoid fractures in Bergen, Norway. Scand J Plast Reconstr Surg Hand Surg.

[4] Steinmann S.P., Adams J.E. (2006). Scaphoid fractures and nonunions: Diagnosis and treatment. J Orthop Sci.

[5] Wong K., von Schroeder H.P. (2011). Delays and poor management of scaphoid fractures: Factors contributing to nonunion. J Hand Surg.

[6] Kawamura K., Chung K.C. (2008). Treatment of scaphoid fractures and nonunions. J Hand Surg.

[7] Brooks S., Cicuttini F.M., Lim S., Taylor D., Stuckey S.L., Wluka A.E. (2005). Cost effectiveness of adding magnetic resonance imaging to the usual management of suspected scaphoid fractures. Br J Sports Med.

[8] Li N.Y., Dennison D.G., Shin A.Y., Pulos N.A. (2023). Update to management of acute scaphoid fractures. J Am Acad Orthop Surg.

[9] Cheng C., Rodner C.M. (2020). Associations between insurance type and the presentation of cubital tunnel syndrome. J Hand Surg.

[10] Menendez M.E., Lu N., Unizony S., Choi H.K., Ring D. (2015). Surgical site infection in hand surgery. Int Orthop.

[11] Sivasundaram L., Wang J.H., Kim C.Y. (2020). Emergency department utilization after outpatient hand surgery. J Am Acad Orthop Surg.

[12] Daniel H., Bornstein S.S., Kane G.C. (2018). Addressing social determinants to improve patient care and promote health equity: An American College of Physicians Position paper. Ann Int Med.

[13] University of Wisconsin School of Medicine and Public Health Area Deprivation Index. https://www.neighborhoodatlas.medicine.wisc.edu/.

[14] Limburg A., Rehkopf D.H., Gladish N., Phillips R.L., Udalova V. (2025). Validating 8 area-based measures of social risk for predicting health and mortality. JAMA Health Forum.

[15] Kind A.J.H., Buckingham W.R. (2018). Making neighborhood-disadvantage metrics accessible—the neighborhood atlas. N Engl J Med.

[16] Dorsay T.A., Major N.M., Helms C.A. (2001). Cost-effectiveness of immediate MR imaging versus traditional follow-up for revealing radiographically occult scaphoid fractures. Am J Roentgenol.

[17] Choo S., Nuelle J.A.V. (2024). Editorial commentary: olecranon bone autografting combined with an arthroscopic approach for the treatment scaphoid nonunion—an important technique for wrist surgeons. Arthrosc J Arthrosc Relat Surg.

[18] Pinder R.M., Brkljac M., Rix L., Muir L., Brewster M. (2015). Treatment of scaphoid nonunion: A systematic review of the existing evidence. J Hand Surg.

[19] Erwin J., Varacallo M.A. (2025). StatPearls.

[20] Hayat Z., Varacallo M.A. (2025). StatPearls.

[21] Sheridan E., Wiseman J., Malik A.T. (2019). The role of sociodemographics in the occurrence of orthopaedic trauma. Injury.

[22] Snell D.T., Lockey P.D., Thompson D.J. (2023). Socioeconomic status is associated with mechanism and intent of injury in patients presenting to a UK Major Trauma Centre. Injury.

[23] Zarzaur B.L., Croce M.A., Fabian T.C., Fischer P., Magnotti L.J. (2010). A population-based analysis of neighborhood socioeconomic status and injury admission rates and in-hospital mortality. J Am Coll Surg.

[24] Yuma-Guerrero P., Orsi R., Lee P.T., Cubbin C. (2018). A systematic review of socioeconomic status measurement in 13 years of U.S. injury research. J Safety Res.

[25] Madsen C., Gabbe B.J., Holvik K. (2022). Injury severity and increased socioeconomic differences: A population-based cohort study. Injury.

[26] Breslin M.A., Bacharach A., Ho D. (2023). Social determinants of health and patients with traumatic injuries: Is there a relationship between social health and orthopaedic trauma?. Clin Orthop.

[27] Mandalia K., Shah S. (2024). Editorial Commentary: The social determinants of health are insufficiently reported in the orthopaedic literature. Arthrosc J Arthrosc Relat Surg.

[28] Bernstein D.N., Lans A., Karhade A.V. (2023). Are detailed, patient-level social determinant of health factors associated with physical function and mental health at presentation among new patients with orthopaedic conditions?. Clin Orthop.

[29] Calfee R.P., Shah C.M., Canham C.D., Wong A.H.W., Gelberman R.H., Goldfarb C.A. (2012). The influence of insurance status on access to and utilization of a tertiary hand surgery referral center. J Bone Joint Surg Am.

[30] Labrum J.T.I., Paziuk T., Rihn T.C. (2017). Does medicaid insurance confer adequate access to adult orthopaedic care in the era of the patient protection and affordable care act?. Clin Orthop Relat Res.

[31] Kaveeshwar S., Hasan S., Polsky D. (2024). Area deprivation index as a proxy for socioeconomic status in outpatient orthopaedic surgery patients—a prospective registry cross sectional study. J Orthop.

[32] Garala K., Taub N.A., Dias J.J. (2016). The epidemiology of fractures of the scaphoid: impact of age, gender, deprivation and seasonality. Bone Joint J.

[33] Zhao H., Tian S., Kong L. (2018). Factors associated with union time of acute middle-third scaphoid fractures: An observational study. Ther Clin Risk Manag.

[34] Murthy N.S. (2013). The role of magnetic resonance imaging in scaphoid fractures. J Hand Surg.

[35] Hsia R.Y., Kellermann A.L., Shen Y.C. (2011). Factors associated with closures of emergency departments in the United States. JAMA.

[36] Simunovic N., Devereaux P.J., Sprague S. (2010). Effect of early surgery after hip fracture on mortality and complications: Systematic review and meta-analysis. CMAJ Can Med Assoc J.

